# IL-36-Driven Inflammation in Generalized Pustular Psoriasis: Immunological Insights from Plaque Psoriasis and Implications for Targeted Therapy

**DOI:** 10.3390/ijms27104441

**Published:** 2026-05-15

**Authors:** Klara Andrzejczak, Emilia Kucharczyk, Agata Sternak, Karol Biliński, Joanna Maj, Małgorzata Ponikowska

**Affiliations:** 1 Faculty of Medicine, Wroclaw Medical University, Wybrzeze L. Pasteura 1, 50-367 Wroclaw, Poland; 2 University Centre of General Dermatology and Oncodermatology, Wroclaw Medical University, 50-556 Wroclaw, Poland; joanna.maj@umw.edu.pl

**Keywords:** generalized pustular psoriasis (GPP), IL-36 signaling, interleukin-36, neutrophils, autoinflammatory disease, plaque psoriasis, IL-23/Th17 axis, targeted therapy, IL-36 receptor inhibition, spesolimab

## Abstract

Generalized pustular psoriasis (GPP) is a rare, severe, and potentially life-threatening inflammatory dermatosis increasingly recognized as a distinct disease entity rather than a variant of plaque psoriasis. Emerging evidence indicates that GPP is primarily driven by dysregulation of the interleukin-36 (IL-36) signaling axis, leading to amplification of proinflammatory cascades in keratinocytes and a predominantly innate, neutrophil-driven immune response. This promotes rapid neutrophil recruitment, sterile pustule formation, and abrupt cutaneous and systemic inflammation. Consistent with this, GPP demonstrates a greater predominance of innate immune and neutrophil-driven inflammation, whereas plaque psoriasis is more strongly associated with IL-23/Th17-mediated adaptive immune responses. Transcriptomic and genetic studies further support this distinction, demonstrating enrichment of IL-36-associated and neutrophil-related signatures, activation of MyD88-dependent pathways, and mutations in genes regulating the IL-36 axis, including *IL36RN*, *AP1S3*, and *CARD14*. Consequently, conventional systemic therapies and biologics targeting TNF-α, IL-17, and IL-23 pathways show variable efficacy and may act more slowly in GPP. In contrast, IL-36 receptor inhibitors represent a more mechanism-aligned approach and have demonstrated rapid and clinically meaningful responses in acute flares. However, important gaps remain, including the lack of validated biomarkers and limited data on long-term treatment outcomes. This review provides an integrated perspective on IL-36-driven inflammation in GPP, including comparison with plaque psoriasis, and outlines its implications for mechanism-based therapeutic approaches.

## 1. Introduction

Psoriasis encompasses a heterogeneous group of chronic inflammatory skin diseases, of which plaque psoriasis is the most common clinical subtype, characterized by well-demarcated, scaly plaques typically involving the extensor surfaces [1]. Pustular psoriasis, in contrast, is a rare clinical entity, accounting for approximately 1% of psoriasis cases and characterized by sterile, neutrophil-rich pustules with a variable distribution pattern [2]. Within this spectrum, generalized pustular psoriasis (GPP) represents the most severe phenotype, characterized by acute, potentially life-threatening episodes with systemic involvement.

Since its first description by von Zumbusch in 1909, the classification of GPP has remained controversial, particularly regarding whether it represents a severe variant of plaque psoriasis or a distinct clinical entity [3,4]. Epidemiological data indicate that GPP is more common in Asian than in Caucasian populations and shows a female predominance, with peak incidence in middle adulthood, although it may occur at any age [4,5,6,7]. Overall, estimates of the prevalence of GPP among patients with psoriasis range from 0.6% to 2.4%, and GPP coexists with plaque psoriasis in a substantial proportion of patients [8,9]. In pediatric populations, the disease is rare but may demonstrate a slight male predominance [10].

According to recent consensus definitions, GPP is recognized as a systemic inflammatory disease characterized by cutaneous erythema and macroscopically visible sterile pustules [11]. The essential diagnostic criterion is the presence of sterile pustules on an erythematous base, not restricted to acral regions or confined to pre-existing psoriatic plaques. GPP may occur with or without systemic symptoms and laboratory abnormalities (such as elevated C-reactive protein, leukocytosis with neutrophilia, and increased erythrocyte sedimentation rate), and can present either as an acute, widespread pustular eruption or as a more subacute, annular phenotype [9,11]. The disease typically follows a relapsing course or may persist for longer than three months, with flares frequently necessitating urgent medical intervention and hospitalization [11,12]. Various endogenous and exogenous factors have been implicated in triggering GPP flares, including infections, pregnancy, psychological stress, abrupt withdrawal of systemic corticosteroids, hypocalcemia, and certain medications such as lithium, terbinafine, or beta-blockers [12]. These triggers may promote excessive activation of innate immune pathways and contribute to rapid amplification of IL-36-driven inflammation in genetically susceptible individuals. Severe episodes may result in life-threatening complications, including sepsis and multi-organ failure [9]. The diagnosis requires exclusion of alternative conditions such as acute generalized exanthematous pustulosis (AGEP), which may show overlapping clinical and histopathological features but is typically associated with acute drug exposure, eosinophilia, and a more self-limited course [11,13].

Histopathologically, GPP is characterized by prominent neutrophilic infiltration within the epidermis, accompanied by acanthosis and parakeratosis, with the formation of spongiform pustules of Kogoj and subcorneal neutrophilic aggregates. However, the National Psoriasis Foundation consensus statement emphasizes that skin biopsy is not essential for the diagnosis of GPP, as histopathological findings may be nonspecific and can overlap with conditions such as AGEP [14].

Historically, GPP has been considered a variant of plaque psoriasis [15,16]. However, increasing clinical, genetic, and immunological evidence supports its classification as a distinct inflammatory disorder. In particular, the identification of mutations in the *IL36RN* gene and related pathways, present in a subset of patients and associated with specific clinical phenotypes, provides strong support for a distinct pathogenic framework [17]. Together with the recognition that GPP may occur independently of plaque psoriasis and exhibits distinct therapeutic responses, this has led to a conceptual shift toward viewing GPP as a predominantly autoinflammatory disease characterized by innate immune dysregulation. Although IL-36 dysregulation represents one of the best-characterized pathogenic mechanisms in GPP, the disease remains clinically and biologically heterogeneous, and its expression likely reflects the interaction of genetic susceptibility, environmental triggers, innate immune activation, and additional inflammatory pathways. Importantly, not all patients carry *IL36RN* mutations, further supporting the heterogeneous nature of the disease. Further studies are needed to better define the relative contribution of these mechanisms to disease heterogeneity and therapeutic response [18,19].

Emerging evidence indicates that GPP is driven predominantly by dysregulation of the interleukin-36 (IL-36) signaling axis, a member of the interleukin-1 (IL-1) cytokine family that plays a central role in innate immune responses [16]. Overactivation of IL-36 signaling leads to amplification of proinflammatory cascades within keratinocytes and promotes the recruitment and activation of neutrophils, resulting in the formation of sterile pustules [20]. These mechanisms highlight fundamental differences in inflammatory organization between GPP and plaque psoriasis, with a predominance of innate immune and neutrophil-mediated processes in GPP compared with adaptive, T cell-driven responses in plaque psoriasis [9,20,21]. Accordingly, therapeutic options for GPP remain limited, and treatment strategies have historically been extrapolated from plaque psoriasis [22].

Conventional systemic therapies and biologics targeting TNF-α, IL-17, and IL-23 pathways show variable and often delayed efficacy, particularly during acute flares [9]. Nevertheless, systemic agents such as retinoids, methotrexate, and cyclosporine, as well as biologics, continue to play an important role in current treatment algorithms for GPP, particularly in maintenance therapy, in patients with concomitant plaque psoriasis, and in settings where IL-36-targeted therapies are not readily available. In contrast, therapies specifically targeting the IL-36 pathway, such as IL-36 receptor inhibitors, represent a mechanism-based approach and have demonstrated rapid and clinically meaningful responses, thereby supporting the central pathogenic role of IL-36 signaling in GPP [20,22,23].

This review aims to characterize IL-36-driven inflammation in the pathogenesis of GPP and to define the disease as a distinct autoinflammatory entity. By comparison with plaque psoriasis, it highlights fundamental differences in inflammatory organization, particularly the relative roles of innate and adaptive immune pathways, with a focus on IL-36-targeted therapies.

## 2. The IL-36 Axis as a Central Pathogenic Pathway in GPP

### 2.1. IL-36 Signaling: Cytokines, Receptor Activation, and Dysregulation

#### 2.1.1. IL-36 Cytokines and Their Expression

The IL-36 cytokine family belongs to the IL-1 superfamily and includes IL-36α, IL-36β, and IL-36γ, which act as pro-inflammatory agonists, as well as the IL-36 receptor antagonist (IL-36Ra), which inhibits IL-36 receptor signaling. In the skin, keratinocytes are the main source of these cytokines, although they can also be produced by immune cells, including macrophages and dendritic cells [15,20,24,25,26,27,28].

IL-36α and IL-36β are present at baseline in healthy skin, whereas IL-36γ expression is low under physiological conditions but increases in psoriatic lesions [20,24]. IL-36 production is induced by cytokines associated with psoriasis pathogenesis, including TNF-α, IL-17, and IL-22. Importantly, IL-36 cytokines can further enhance IL-36 gene expression in keratinocytes, leading to a positive feedback loop and a self-amplifying inflammatory process [15,29,30]. Increased IL-36 expression has also been confirmed at the mRNA level in psoriatic skin lesions [15,29].

#### 2.1.2. IL-36 Receptor Signaling Pathways

**Figure 1:**
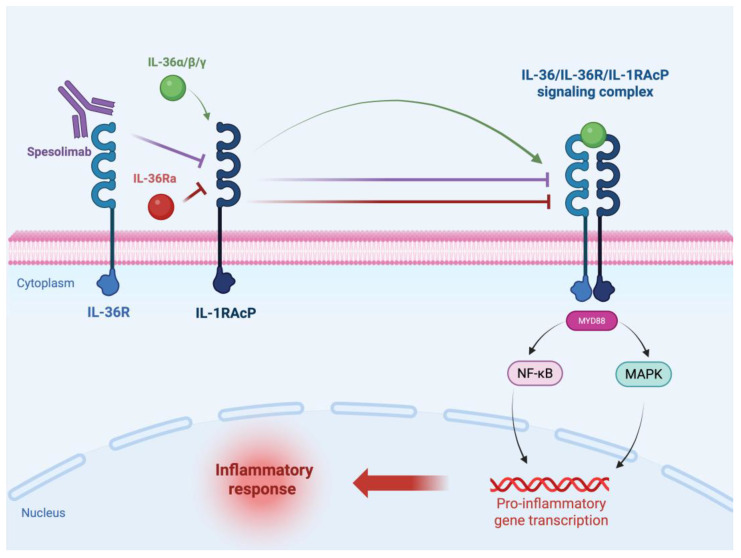
IL-36 receptor signaling and receptor complex formation. IL-36 agonists induce formation of the IL-36R/IL-1RAcP signaling complex, leading to activation of NF-κB and MAPK pathways. IL-1RAcP functions as an essential co-receptor required for signal transduction. The IL-36Ra antagonist competitively inhibits this process by preventing IL-1RAcP recruitment. Spesolimab blocks IL-36R, thereby inhibiting downstream signaling. Created in BioRender. Andrzejczak, K. (2026) https://BioRender.com/nep8sxa (accessed on 13 May 2026). Abbreviations: IL-36α/β/γ—Interleukin-36 alpha/beta/gamma, IL-36Ra—Interleukin-36 receptor antagonist, IL-36R—Interleukin-36 receptor, IL-1RAcP—Interleukin-1 receptor accessory protein, MyD88—Myeloid differentiation primary response 88, NF-κB—Nuclear factor kappa-light-chain-enhancer of activated B cells, MAPK—Mitogen-activated protein kinase.

IL-36 agonists bind to the IL-36 receptor (IL-36R, also known as IL1RL2 or IL-1Rrp2), a membrane receptor composed of three extracellular domains and one intracellular Toll/interleukin-1 receptor (TIR) domain. Ligand binding promotes the recruitment of IL-1RAcP, a key co-receptor required for signal transduction, resulting in the formation of a ternary (IL-36/IL-36R/IL-1RAcP) signaling complex. This leads to the association of the intracellular TIR domains, thereby initiating MyD88-dependent signaling involving mitogen-activated protein kinases (MAPK) and nuclear factor κB (NF-κB)-dependent transcription. These pathways ultimately drive a pro-inflammatory response, as schematically illustrated in Figure 1 [15,20,24,31,32,33].

Conversely, the IL-36Ra antagonist competes for binding to the IL-36 receptor, thereby preventing the recruitment of IL-1RAcP and inhibiting downstream signaling. These findings underscore the central role of IL-36 signaling in GPP pathogenesis and support targeting this pathway as a mechanism-based therapeutic strategy [20,24,25,31].

However, several aspects of IL-36 biology remain incompletely understood, including the temporal regulation of pathway activation and resolution and the mechanisms that restrict excessive signaling. Potential compensatory interactions within the IL-1 cytokine family, as well as the influence of the cutaneous microenvironment, in which keratinocytes and immune cells form dynamic inflammatory circuits, may further modulate IL-36-driven responses in a context-dependent manner. In addition, the differential roles of IL-36 isoforms (IL-36α, IL-36β, and IL-36γ), together with their proteolytic activation by neutrophil- and keratinocyte-derived proteases, introduce an additional layer of complexity that remains incompletely determined [32,34,35].

#### 2.1.3. IL-36 Dysregulation in GPP

Under physiological conditions, the activity of IL-36 agonists is balanced by high levels of IL-36Ra. However, disruption of this balance, for example due to increased expression of IL-36 agonists or reduced activity of IL-36Ra, such as in the presence of *IL36RN* mutations, leads to an excessive and uncontrolled pro-inflammatory response [15,24].

Mutations in the *IL36RN* gene result in a deficiency of the IL-36 receptor antagonist, manifesting as an autoinflammatory disease known as DITRA. This condition typically presents in childhood and is characterized by recurrent and severe episodes of GPP [24]. The frequency of *IL36RN* mutations in patients with GPP ranges from 10% to 82%. This variability reflects differences in study populations, including ethnic background, clinical phenotype, age of onset, and the presence or absence of concomitant plaque psoriasis. It may also be influenced by variations in diagnostic criteria, sample size, and potential selection bias [20]. Mutation rates differ across populations. Higher frequencies are reported in East Asian cohorts, whereas lower rates are observed in multi-ethnic populations. These differences likely reflect both genetic background and clinical heterogeneity, including earlier disease onset and a higher likelihood of GPP without concomitant plaque psoriasis in mutation-positive patients [18,36,37].

In addition, *IL36RN* mutations are more frequently observed in patients with GPP without concomitant plaque psoriasis compared to those with overlapping plaque psoriasis [18,38]. Importantly, a substantial proportion of patients with GPP do not carry identifiable *IL36RN* mutations, indicating that these mutations are not required for disease development and highlighting the genetic heterogeneity of GPP and the contribution of additional pathogenic mechanisms [39].

In a study by Swindell et al., stimulation of keratinocytes with IL-36 was shown to induce changes in the expression of genes associated with the inflammatory response. These include increased expression of pro-inflammatory cytokines such as *IL1A, IL1B*, and *IL36G*, as well as genes involved in inflammatory processes, including those encoding proteases such as *CTSS* [40]. Furthermore, dysregulation of IL-36 signaling may be associated not only with loss-of-function mutations in *IL36RN*, but also with alterations in genes related to this pathway, such as *CARD14*, *AP1S3*, and *SERPINA3*. These changes lead to activation of NF-κB signaling pathways and further upregulation of cytokines, including CXCL1-3, IL-1, IL-8, and IL-36 [20,21].

These findings highlight the relationship between genetic determinants and clinical presentation in pustular psoriasis, as summarized in Table 1.

Importantly, isolated GPP and GPP associated with plaque psoriasis may differ in their underlying immunological mechanisms. Isolated GPP is more strongly associated with *IL36RN* mutations and IL-36 dysregulation, whereas concomitant plaque psoriasis may involve greater contribution of the IL-23/IL-17 axis, which could also influence therapeutic choice [12,18].

An imbalance between IL-36 agonists and IL-36Ra promotes the persistence of inflammation in the skin and represents a key factor driving the development of generalized pustular psoriasis. These findings highlight the pivotal role of IL-36 dysregulation in disease pathogenesis and support targeting this pathway as a mechanism-based therapeutic strategy [24,46].

### 2.2. Neutrophil-Dependent Activation of the IL-36 Axis

Neutrophils play a crucial role in the elimination of pathogens through degranulation, production of reactive oxygen species (ROS), phagocytosis, and the formation of neutrophil extracellular traps (NETs) [47,48]. Their presence at sites of inflammation serves not only a protective function by neutralizing microorganisms, but also contributes to shaping and amplifying the inflammatory response through modulation of pro-inflammatory cytokines. In response to infection or tissue damage, alarmins, including IL-36 and other cytokines of the IL-1 family, are released into the extracellular space [19,35].

IL-36 cytokines are released as inactive precursors and require proteolytic processing to achieve full biological activity. This process is mediated by neutrophil-derived proteases, such as cathepsin G, proteinase 3, and elastase, as well as cathepsin S derived from keratinocytes, resulting in up to a 500-fold increase in their activity [15,19,24,25,29,35].

Neutrophil proteases exhibit substrate specificity toward IL-36 isoforms. IL-36α is processed and activated by cathepsin G and elastase, IL-36β is selectively activated by cathepsin G, whereas IL-36γ is activated by elastase and proteinase 3 [35,49,50].

This processing involves the removal of a small number of amino acid residues from the N-terminus of the molecule [15,35]. To regulate the inflammatory response, keratinocytes produce endogenous serine protease inhibitors, such as serpin A1 and serpin A3, which inhibit neutrophil proteases; however, these mechanisms are not sufficient to fully control this inflammatory axis [29].

These findings indicate that neutrophils function not only as effector cells of the innate immune response, but also actively enhance IL-36-dependent inflammation by increasing the bioactivity of these cytokines [19,24,35].

### 2.3. The IL-36-Driven Keratinocyte–Neutrophil Loop in GPP

IL-36 is upregulated in skin lesions of GPP [29]. Excessive activation of the IL-36 receptor leads to stimulation of keratinocytes as well as myeloid dendritic cells, resulting in enhanced production of pro-inflammatory mediators. In keratinocytes, IL-36 induces the production of neutrophil-attracting chemokines, such as CXCL1, CXCL2, and CXCL8 (IL-8), and promotes activation of the Th17/IL-23 axis, leading to neutrophil accumulation and psoriasiform inflammation [20,24,25,29,31].

Neutrophils constitute the dominant population of leukocytes in skin lesions of pustular psoriasis. Recruited cells release proteases responsible for IL-36 activation through proteolytic processing, as well as neutrophil extracellular traps (NETs), which further contribute to IL-36 activation [25,51]. Neutrophil-derived proteases activate IL-36 cytokines, thereby enhancing their ability to stimulate keratinocytes to produce neutrophil-attracting chemokines [29]. Consequently, IL-36-dependent signaling is further amplified, leading to sustained activation of keratinocytes and increased production of pro-inflammatory mediators [24,46,51]. Collectively, neutrophils contribute to the amplification of a positive IL-36-driven autoinflammatory loop, promoting the escalation of inflammation in GPP [25]. Increased expression of IL-36α and IL-36γ in skin samples and peripheral blood further supports the central role of IL-36 dysregulation in this disease [24].

Chronic activation of IL-36-dependent signaling, together with sustained neutrophil recruitment, leads to their accumulation in the skin, which is a characteristic feature of GPP. This results in the formation of sterile pustules, including spongiform pustules of Kogoj and subcorneal neutrophil accumulations referred to as ‘lakes of pus’ [19,20,21]. The disease is characterized by recurrent flares, often accompanied by systemic symptoms such as fever, malaise, and pain [21,24].

The self-amplifying nature of the IL-36-neutrophil axis may contribute to rapid amplification of inflammation, driving the progression of GPP skin lesions and the development of systemic manifestations [24].

Environmental and clinical triggers, including infections, withdrawal of corticosteroids, pregnancy, stress, and certain medications, may precipitate GPP flares and further amplify IL-36-mediated inflammation [12,20]. These factors are thought to enhance innate immune activation and neutrophil recruitment, thereby contributing to the initiation or exacerbation of GPP episodes [20,24].

## 3. Comparative Insights from Plaque Psoriasis

### 3.1. Immune Architecture and Interplay Between IL-36 and IL-23/Th17 Pathways

Although the IL-36-driven inflammatory cascade represents the central pathogenic mechanism in GPP, its immunological phenotype differs in its dominant immunological features from that observed in plaque psoriasis. Plaque psoriasis is currently understood as a T cell-driven inflammatory disease, primarily involving the IL-23/Th17 axis, with additional contributions from Th1 and Th22 cells [52].

In this condition, T cells orchestrate the inflammatory response through interactions with multiple cell populations, including dendritic cells, neutrophils, and keratinocytes. The role of adaptive immunity is well established in plaque psoriasis, as activated Th cells produce key cytokines such as IL-17A, IL-17F, IL-22, and IFN-γ, which promote keratinocyte proliferation [53].

It has also been shown that IL-17A triggers the production of IL-36 cytokines in keratinocytes [54]. In turn, IL-36 synergizes with IL-17A to enhance the expression of pro-inflammatory genes, creating a positive feedback loop that amplifies inflammation [54]. This interaction is reflected in current therapeutic strategies, where IL-17A inhibitors demonstrate high efficacy in plaque psoriasis [55,56,57], whereas IL-36-targeted therapies have no established role in its treatment.

By comparison, GPP is characterized by dysregulation of IL-36 signaling, which plays a central role in shaping its immunological profile. In GPP, keratinocytes act as a major source of IL-36. This drives a predominantly innate immune response, while also engaging adaptive pathways and promoting neutrophil recruitment and activation [58]. Such activity underscores the role of keratinocytes as central regulators of cutaneous immunity; by integrating innate and adaptive signals through the secretion of diverse chemokines, they directly influence the migration and functional states of T cells, neutrophils, and dendritic cells [59]. This neutrophil-driven inflammation subsequently activates dendritic cells to release TNF-α and IL-23, creating a feedback loop that enhances IL-36 production and supports Th17 differentiation. Ultimately, this highlights a functional interplay where IL-36-driven inflammation not only initiates neutrophil recruitment but also fuels the downstream activation of the IL-23/Th17 axis.

These mechanisms align with clinical observations, as IL-17A inhibitors have shown clinical efficacy in selected patients; however, treatment outcomes remain variable and are supported by limited evidence [60,61,62,63].

Importantly, a significant proportion of patients with GPP present with concomitant plaque psoriasis, suggesting overlapping clinical and immunological pathways [8,58]. This overlap may reflect a shared pathway involving the IL-17/IL-23 mediated adaptive response, while IL-36-driven innate inflammation remains dominant in GPP [20,24,58]. The coexistence of plaque psoriasis likely contributes to the heterogeneity of clinical presentation and therapeutic response. This might explain why some patients respond better to IL-17 or IL-23 inhibition despite the central role of IL-36 in pathogenesis of GPP [18,61,62,63,64].

Collectively, these observations underscore a key distinction in the hierarchy of inflammatory pathways, with IL-23/Th17-mediated adaptive immunity predominant but not exclusive in plaque psoriasis and IL-36-mediated innate responses dominating in GPP.

### 3.2. Transcriptomic Signatures Distinguishing GPP and Plaque Psoriasis

Immunological differences between GPP and plaque psoriasis are also reflected at the transcriptomic level, where distinct gene expression profiles further support their divergent pathogenesis. Transcriptomic analyses, including single-cell and spatial RNA sequencing, have demonstrated enhanced communication between *IL36G+* keratinocytes and neutrophils [64]. Furthermore, the study demonstrated a characteristic shift from caspase-8 (*CASP8*)-positive to *CASP8*-negative neutrophils. Given that *CASP8* has been shown to prevent necroptosis and thereby limit inflammatory responses, its loss may promote cell death-associated inflammation, providing new insights into GPP pathogenesis [64,65].

In addition, transcriptomic data revealed increased expression of *TNFSF15*, encoding TNF-like ligand 1A (TL1A), particularly in neutrophils. TL1A, a member of the TNF family, acts as an amplifier of inflammatory signaling and promotes Th17-related cytokine production, highlighting a link between innate and adaptive immunity in GPP [66]. The elevated expression of *TNFSF15* in neutrophils points to unconventional inflammatory pathways that could be modulated via monoclonal antibodies [64].

In a study by Swindell et al., IL-36 was shown to induce a MyD88-dependent transcriptional program in keratinocytes [40]. Consistent with these findings, IL-36-induced gene expression signatures show stronger enrichment in GPP lesions compared with plaque psoriasis. Mechanistically, IL-36 signaling leads to the upregulation of pro-inflammatory cytokines, proteases, and key signaling components, including MyD88. Moreover, IL-36 signaling increases IL-23A expression, providing a functional link between innate immune activation and the IL-23/Th17 axis. Importantly, inflammatory pathway-related genes, such as *IL1B*, *CTSL*, and *CD14*, which are involved in MAPK and NF-κB signaling, are downregulated following treatment with acitretin and glucocorticoids, further supporting their role in GPP pathogenesis [67].

Transcriptomic studies of plaque psoriasis have shown that gene expression profiles are often shared with other inflammatory skin diseases [68]. In addition to psoriasis-specific genes, such as *ATP1B1*, *PRR9*, and *PON2*, which are preferentially induced by IL-17A, non-specific genes, including *S100A8*, *IFI27*, and *STAT1*, are primarily regulated by IFN-γ and TNF [68].

Although still not fully understood, alternative splicing represents an additional layer of transcriptomic regulation in psoriasis [69,70,71]. A notable example of the functional consequences of alternative splicing is the *TRAF3IP2* gene. An isoform lacking exon 2 impairs IL-17 signal transduction; paradoxically, this defect promotes overproduction of pro-inflammatory cytokines, including IL-17 and IL-22, thereby increasing disease susceptibility. In contrast, specific IL-15 isoforms, including the exon 7-excluded variant, may exert protective effects by limiting keratinocyte proliferation and reducing dermal neutrophil infiltration [70,72].

Additional transcriptomic evidence from Catapano et al. demonstrated that patients with GPP exhibit higher IFN scores, a composite measure used to quantify the aggregate expression of *IFI6*, *IFIT3*, *IFITM3*, *OASL*, and *PLSCR1*, compared with those with plaque psoriasis [73]. This finding reflects a stronger type I interferon-driven molecular signature in GPP. Among IFN-induced genes, IFI6 encodes interferon alpha-inducible protein 6, which acts as an anti-apoptotic protein in proliferating keratinocytes. Its upregulation promotes keratinocyte hyperproliferation and reduced apoptosis, thereby contributing to the epidermal thickening observed in psoriasis [74]. Importantly, individuals with higher IFN scores are more prone to systemic flares, suggesting that the IFN-I/IFI6 axis contributes to both disease severity and systemic inflammation. Mechanistically, data suggests that IL-36 amplifies IFN-I responses by activating plasmacytoid dendritic cells. These cells express high levels of IL-36R and drive IRF7-dependent IFN-I production. This interaction likely accounts for the IFN-I signatures observed in GPP, explaining its systemic clinical phenotype as distinct from plaque psoriasis [73].

### 3.3. Genetic Distinctions Supporting Biological Divergence

Current evidence supports the concept that GPP and plaque psoriasis have distinct biological bases. Plaque psoriasis is a multifactorial disease, with genetic factors accounting for approximately 70% of susceptibility [75]. The strongest genetic association lies within the major histocompatibility complex (MHC) at the PSORS1 locus, particularly the HLA-C*06:02 allele [20].

While other loci, such as *CCHCR1* and *CDSN*, have been associated with psoriasis, their roles remain incompletely understood. Mechanistically, HLA-C*06:02 may exhibit higher affinity for psoriasis autoantigens, such as the specific melanocyte autoantigen (ADAMTS-like protein 5) and LL-37, both described as T-cell autoantigens important in psoriasis. Although HLA-C*06:02 remains the strongest genetic driver, additional independent risk factors have been identified. Amino acid positions 67 and 9 in HLA-B, position 95 in HLA-A, and position 53 in HLA-DQα1 show stronger associations with disease susceptibility than classical alleles [76].

Furthermore, distinct HLA loci contribute to both overall susceptibility and phenotypic heterogeneity; for example, variation at HLA-B position 45 has been associated with an increased risk of psoriatic arthritis [76].

Beyond the MHC region, genome-wide association studies (GWAS) have identified numerous non-MHC susceptibility loci [77]. In European populations, variants in *IL12B*, *IL23R*, and *TRAF3IP2* show significant associations with psoriasis susceptibility, whereas studies in non-European populations have highlighted *IL12B*, *LCE3A*, and *LCE3D* as key loci associated with plaque psoriasis [77]. Overall, these findings indicate that plaque psoriasis arises from the convergence of immune and epidermal pathways shaped by both shared and population-specific genetic factors.

In contrast, GPP is strongly associated with mutations in the *IL36RN* gene. A landmark study by Rachid Marrakchi et al. identified a substitution of leucine with proline at position 27 of the IL-36Ra [22]. Biochemical analyses suggest that this alteration reduces its ability to bind the IL-36 receptor (IL-1Rrp2), thereby failing to inhibit pro-inflammatory signaling and leading to excessive cytokine production. Subsequent studies have shown that *IL36RN* mutations are common in GPP and absent in plaque psoriasis. Notably, their prevalence varies across clinical phenotypes, occurring in up to 82% of patients with isolated GPP compared with approximately 17% of those with concomitant plaque psoriasis [78].

In contrast, the major plaque psoriasis susceptibility allele, HLA-C*06:02, is not associated with GPP. Moreover, *IL36RN* mutations may exhibit incomplete penetrance, requiring specific environmental triggers or modifier genes to manifest clinically. For example, some individuals with homozygous frameshift mutations remain asymptomatic until late adulthood, with disease onset occurring after the age of 65 years [44].

Another important genetic alteration associated with GPP involves *AP1S3*, which can be inherited in conjunction with *IL36RN* [43]. *AP1S3* mutations impair keratinocyte autophagy, leading to accumulation of p62 and enhanced NF-κB activation, thereby promoting cytokine production. Additional genetic contributors include gain-of-function mutations in *CARD14*. Unlike *IL36RN* mutations, which are predominantly linked to isolated GPP, *CARD14* variants frequently associate with a mixed phenotype. Indeed, a significant proportion of GPP patients carrying *CARD14* mutations also present with concomitant plaque psoriasis [79].

In addition, variants in MPO have also been linked to pustular psoriasis rather than strictly GPP, where they may contribute to disease by reducing neutrophil apoptosis and prolonging cell survival [80].

Beyond the primary drivers, several other loci have been linked to GPP, though their roles remain less clear due to limited study populations: *SERPINA3* (impairs IL-36 inhibition), *BTN3A3* (triggers excessive NF-κB signaling), and *MEFV* (activates the pyrin inflammasome) [79].

Although these variants affect distinct molecular pathways, they frequently culminate in shared inflammatory mechanisms, such as enhanced NF-κB signaling, neutrophil activation, and the amplification of IL-36-dependent inflammation. *IL36RN* mutation directly impairs IL-36 antagonism, whereas *AP1S3* and *CARD14* promote keratinocyte stress responses and NF-κB activation. Meanwhile, *MPO* and *MEFV* variants primarily affect neutrophil survival and inflammasome signaling. Collectively, these findings support the concept that genetically heterogeneous alterations ultimately manifest as a common IL-36-centered phenotype [20,21,24,79].

Taken together, these findings highlight fundamental differences in inflammatory organization, transcriptomic signatures, and genetic background between plaque psoriasis and GPP, as summarized in Table 2.

## 4. Therapeutic Implications of IL-36 Pathway Activation in GPP

### 4.1. Limitations of Conventional Psoriasis Biologics in GPP

Due to differences in the dominant immune architecture and inflammatory pathways between plaque psoriasis and GPP, conventional psoriasis therapies show variable efficacy and may exhibit a delayed onset of action, particularly during acute flares [91].

Treatment approaches based on retinoids, cyclosporine, or methotrexate may be effective in selected cases of GPP particularly in non-acute disease or as maintenance therapy; however, they are associated with significant toxicity and are frequently insufficient in more severe disease [87,92,93]. Consequently, biologic therapies may represent an important therapeutic option in selected patients. In GPP, biologics targeting IL-17 (brodalumab, ixekizumab, secukinumab), TNF-α (adalimumab, certolizumab), IL-23 (guselkumab, risankizumab), and IL-12/23 (ustekinumab) have been used [87].

A key limitation of these therapies is their variable efficacy. While some patients demonstrate favorable responses, the available evidence is largely based on small studies, case series, or reports, which may overrepresent positive outcomes and limit the ability to determine the true proportion of responders [94].

Moreover, supporting data are derived primarily from small or uncontrolled studies, and study populations are often restricted to single-country cohorts, limiting generalizability [92,95]. Differences in study design further complicate direct comparisons. Additionally, the relapsing-remitting nature of GPP complicates patient selection and outcome assessment in clinical trials [87,92,94,95].

Another limitation is the relatively slow onset of action of these agents, which is suboptimal in a disease characterized by acute flares requiring rapid disease control [87].

These limitations likely reflect differences in the underlying immunopathological mechanisms between GPP and plaque psoriasis. While the IL-23/IL-17 axis predominates in plaque psoriasis, GPP is primarily driven by dysregulated IL-36 signaling and neutrophil-mediated autoinflammatory responses [24,30].

Importantly, these therapies may still play a role in specific clinical contexts, including non-acute disease, maintenance treatment, limited access to IL-36-targeted therapies, or in patients with concomitant plaque psoriasis [12].

Collectively, these observations suggest that currently used biologic therapies may have limited effectiveness in GPP due to their misalignment with the dominant disease mechanisms. This underscores the need for GPP-specific therapies capable of providing rapid control of acute flares and sustained, relapse-free remission [94].

### 4.2. Clinical Evidence for IL-36 Blockade as a Mechanism-Based Therapeutic Strategy

Recognition of IL-36 pathway involvement in GPP has led to the development of targeted therapeutic strategies, including monoclonal antibodies directed against the IL-36 receptor. Currently, these include spesolimab and imsidolimab. Spesolimab is a first-in-class humanized immunoglobulin G1 monoclonal antibody that acts as an antagonist of the IL-36 receptor, thereby inhibiting the downstream inflammatory response. It has been approved by both the FDA and EMA for the treatment of GPP flares in adults [24,88,92,96,97]. It provides rapid and clinically meaningful improvements in pustular clearance and symptom control during acute flares, with a favorable safety profile. In addition, clinical studies are ongoing to evaluate its use in other immune-mediated diseases, including ulcerative colitis, Crohn’s disease, palmoplantar pustulosis, and hidradenitis suppurativa [88,96,98,99,100].

The efficacy and safety of spesolimab were initially evaluated in an open-label phase I study involving seven adult patients with moderate-to-severe GPP flares who had not previously received biologic therapy. Three patients were found to carry homozygous *IL36RN* mutations, and one of them also had a heterozygous mutation in *CARD14*. Treatment response was assessed using the Generalized Pustular Psoriasis Physician Global Assessment (GPPGA), where a score of 0/1 indicates clear or almost clear skin. Detailed results are presented in Table 3 [89,92,96]. Clinical responses were sustained for up to 20 weeks, and no serious adverse events were reported [89,92].

Subsequently, phase II clinical trials, collectively known as the EFFISAYIL program, were conducted, comprising three studies: EFFISAYIL-1, EFFISAYIL-2, and EFFISAYIL-ON [92].

#### 4.2.1. EFFISAYIL-1: Phase II Randomized Controlled Trial

The EFFISAYIL-1 study was a randomized, double-blind, placebo-controlled, multicenter phase II trial. A total of 53 patients with moderate-to-severe GPP were enrolled, including 7 patients with *IL36RN* mutations. Participants were randomized in a 2:1 ratio, with the larger group receiving a single intravenous dose of 900 mg of spesolimab and the smaller group receiving placebo [24,90,92,96,101,102]. Week 1 results are presented in Table 4 [92].

On day 8, patients in the placebo group were allowed to receive a rescue dose of spesolimab, while those in the spesolimab group could receive an additional 900 mg dose. Among the 49 patients followed for 12 weeks, 6 experienced a flare requiring an additional dose [24,90,92,96].

Patients were also assessed using the Visual Analog Scale (VAS), Psoriasis Symptom Score (PSS), Functional Assessment of Chronic Illness Therapy-Fatigue (FACIT-F), and Dermatology Life Quality Index (DLQI). During the first week of treatment, greater improvements were observed in the spesolimab group; however, after day 8, outcomes became comparable between groups [92].

The results suggest that the clinical response and safety profile of spesolimab are independent of sex, body weight, race, and concomitant medications. The presence of *IL36RN* mutations did not preclude a clinical response, although a faster onset of response was observed in these patients [24,92].

Spesolimab was generally well tolerated, and most adverse events were mild to moderate in severity [92,102]. Detailed safety data are presented in Table 5. Adverse events did not lead to treatment discontinuation. The most serious adverse events included a severe urinary tract infection and two cases of DRESS [92].

These findings demonstrate that spesolimab provides superior efficacy compared with placebo while maintaining a favorable safety profile in the treatment of GPP flares [24,92].

#### 4.2.2. EFFISAYIL-2: Prevention of GPP Flares

EFFISAYIL-2 was a multicenter, international, randomized, double-blind, placebo-controlled phase IIb trial designed to evaluate the efficacy of spesolimab in preventing GPP flares and maintaining disease control [92,96,103].

The study included 123 patients aged 12 to 75 years. Participants were randomized in a 1:1:1:1 ratio to receive different loading and maintenance doses of spesolimab or placebo. Treatment lasted 44 weeks, followed by a 4-week follow-up period. The primary endpoint was time to first flare. Detailed results are presented in Table 6 [92,103,104].

Treatment with spesolimab was shown to reduce the frequency of GPP flares compared with placebo. A dose–response relationship was observed, with the most pronounced benefit in the high-dose group, whereas lower doses did not demonstrate statistically significant differences compared with placebo [92,96,103,104]. Moreover, spesolimab reduced the risk of worsening PSS and DLQI scores; however, statistical significance was achieved only for DLQI in the high-dose group [92].

The safety profile of spesolimab was comparable to placebo, and the incidence of adverse events was not dose-dependent. Most events were mild to moderate in severity. Serious adverse events occurred more frequently in patients receiving spesolimab compared with placebo. Adverse events leading to treatment discontinuation were reported in 5.4% of patients. The most commonly reported adverse events are presented in Table 7 [92].

#### 4.2.3. EFFISAYIL-ON: Long-Term Extension Study

To evaluate the long-term safety and efficacy of spesolimab, patients who completed the EFFISAYIL-1 or EFFISAYIL-2 trials were eligible to participate in the open-label, 5-year extension study EFFISAYIL-ON [92,96]. The primary endpoint is the assessment of adverse events up to week 252 of maintenance treatment with spesolimab. Key secondary endpoints include the evaluation of disease relapses, defined based on GPPGA score, as well as the time required to achieve clinical response (GPPGA 0/1) following rescue therapy [92]. The estimated primary completion date is September 2027, with study completion expected in April 2028 [105].

Interim data from the EFFISAYIL-ON extension study suggest sustained clinical efficacy and a safety profile comparable to that observed in previous trials. Long-term subcutaneous treatment was associated with reduced flare frequency, with most patients remaining flare-free during follow-up. The annualized flare rate also declined compared with the pre-treatment period, supporting durable disease control. Importantly, no new safety signals were identified during prolonged exposure, further supporting the favorable long-term safety profile of spesolimab. However, results for the primary endpoint at week 252 are still awaited [106].

Spesolimab also demonstrates immunogenic potential, with anti-drug antibodies (ADAs) detected in some patients following treatment. However, the impact of these antibodies on safety and efficacy has not been fully established. Available data suggest that therapeutic efficacy is maintained despite the presence of ADAs or neutralizing antibodies [92,107].

Despite promising clinical results confirming superiority over placebo, further evaluation of long-term safety, efficacy, and immunogenicity is warranted. Future multicenter, open-label phase IV studies will be important to better define the role of spesolimab in the management of relapsing GPP. The ongoing EFFISAYIL-ON study is expected to provide additional insights into long-term safety and treatment durability, which will be essential for optimizing therapeutic strategies [92,102].

While the EFFISAYIL clinical program provides robust evidence supporting the efficacy of spesolimab in GPP, several limitations should be considered. Study populations remain relatively small due to the rarity of the disease, and patient cohorts are heterogeneous in terms of clinical presentation and genetic background. In EFFISAYIL-1, the use of early rescue therapy and the lack of active comparators limit the assessment of sustained efficacy and comparative effectiveness. In addition, the generalizability of these findings and the long-term impact of immunogenicity require further investigation [90,92,103].

### 4.3. Emerging IL-36 Inhibitors

Another monoclonal antibody targeting the IL-36 receptor is imsidolimab [15]. It is a humanized IgG4 monoclonal antibody with high affinity for the IL-36 receptor [96].

Efficacy, safety, and tolerability were evaluated in the GALLOP study. This study included 8 patients who received a single intravenous dose of imsidolimab on day 1, followed by additional doses on days 29, 57, and 85. Clinical response was assessed using the Clinical Global Impression (CGI) scale. At weeks 4 and 16, clinical response was achieved in 75% of patients, demonstrating the drug’s efficacy in GPP. Moreover, DLQI scores decreased compared with baseline. Adverse events occurred in 75% of patients, with severe events reported in 2 individuals [15,96,108,109].

Further evaluation of imsidolimab included two randomized, double-blind, placebo-controlled phase III trials: GEMINI-1 and GEMINI-2 [109]. In the GEMINI-1 study, 45 patients were enrolled, and 53.3% of those receiving the antibody achieved clear or almost clear skin (GPPGA 0/1) by week 4, compared with 13.3% in the placebo group [96,109].

In GEMINI-2, 16 patients who responded to treatment in GEMINI-1 were randomized to receive monthly maintenance dosing for at least 24 weeks. All patients receiving maintenance therapy maintained a GPPGA 0/1 response throughout the study period, whereas only 25% of patients in the placebo group achieved this outcome [109].

Imsidolimab demonstrates a favorable safety profile. No serious adverse events were reported, and the incidence of infections was low. Anti-drug antibodies were detected in only one patient [96,109].

The available evidence suggests that imsidolimab represents a promising therapeutic option for the treatment of GPP; however, it has not yet been approved for clinical use. Limitations include the small number of studies and relatively small patient populations. Further research is required to confirm its long-term efficacy and safety. Targeting key inflammatory pathways, such as IL-36 signaling, holds significant potential to improve disease management in GPP. Overall, these findings are based on relatively small patient cohorts, which may limit their generalizability [108,109].

### 4.4. Clinical Application and Comparative Perspective of IL-36 Inhibitors

In clinical practice, IL-36 inhibitors are primarily used for the rapid control of acute GPP flares, where timely intervention is critical [90,92]. Emerging evidence also suggests a potential role in relapse prevention and long-term disease control, although optimal maintenance strategies and retreatment approaches have not yet been clearly defined [90,92,96]. Their high efficacy and rapid onset of action suggest promising future applications not only in flare control but also in the maintenance of disease remission. However, further long-term studies are needed to better define their role in maintenance therapy [90,103].

A comparative overview of key characteristics of IL-36 receptor inhibitors currently investigated in GPP is presented in Table 8.

However, direct comparisons between spesolimab and imsidolimab remain limited due to differences in study design, patient populations, and evaluated endpoints, as well as the absence of head-to-head clinical trials [92].

Available evidence suggests that IL-36 inhibitors may provide a more rapid onset of action than conventional biological therapies, particularly during acute flares; however, definitive conclusions regarding their comparative effectiveness cannot yet be established [24,90].

The use of IL-36 inhibitors may depend on disease severity, flare frequency, prior treatment response, and patient-specific factors [24]. Patient selection for IL-36-targeted therapy may be guided by clinical features such as acute, severe GPP flares requiring rapid disease control, frequent relapses, inadequate response or intolerance to conventional systemic or biological therapies, and limited availability of effective alternative treatment options [90,92,103].

Importantly, evidence regarding the use of IL-36 inhibitors in special populations, including children, adults older than 75 years, pregnant women, and patients with multiple comorbidities, remains limited because these groups have been underrepresented or excluded from clinical trials [92].

In addition, practical considerations such as cost, access to therapy, and healthcare system constraints may influence their real-world use [103,109].

Overall, IL-36 inhibition represents a promising mechanism-based therapeutic strategy in GPP; however, further long-term and real-world studies are needed to better define its optimal place in clinical practice [92].

## 5. Translational Insights and Biomarker Development in GPP

### 5.1. Genetic Biomarkers

Genetic studies have established *IL36RN* mutations as a model of IL-36-driven disease, providing important insights into the central role of this pathway in GPP [18,110]. However, the majority of patients do not carry *IL36RN* variants, indicating that IL-36 pathway activation represents a broader pathogenic mechanism independent of mutation status [18].

Importantly, *IL36RN* mutations have been associated with specific clinical features, including earlier disease onset and distinct phenotypes such as hyponychial pustulation. Nevertheless, their clinical utility as predictive biomarkers remains limited, as they do not appear to reliably correlate with therapeutic response to acitretin or recurrence frequency, highlighting the multifactorial nature of disease expression and the contribution of additional genetic and environmental factors [111].

Although *IL36RN* mutations provide important mechanistic insights and are associated with selected clinical phenotypes, their clinical applicability as predictive biomarkers remains limited. At present, no genetic biomarker has demonstrated sufficient sensitivity or specificity to reliably predict disease course, relapse risk, or therapeutic response in routine clinical practice.

### 5.2. Transcriptomic Biomarkers

Transcriptomic analyses further support the central role of innate immune activation in GPP and provide additional insight into potential biomarkers and therapeutic targets. RNA sequencing studies have demonstrated that genes associated with neutrophil recruitment and activation, including *CXCL1*, *CXCL8*, *S100A8*, *S100A9*, and *LCN2*, are significantly downregulated during clinical remission, paralleling therapeutic response. These findings highlight a strong enrichment of neutrophil-related and inflammatory pathways in active disease, while also identifying novel regulatory networks and upstream mediators, such as oncostatin M, as potential targets for future intervention [112].

These findings support the potential utility of transcriptomic profiling in identifying disease-associated inflammatory pathways and therapeutic targets. However, the availability, cost, and technical complexity of transcriptomic analyses currently limit their routine clinical application.

### 5.3. Serum Inflammatory Biomarkers

Ongoing efforts to identify molecular biomarkers in inflammatory skin diseases aim to improve diagnosis, assess disease severity, and guide treatment selection. Several circulating biomarkers reflecting systemic inflammation and disease activity have been investigated in GPP.

While genetic markers such as HLA-C*06:02 have demonstrated predictive value in plaque psoriasis, analogous biomarkers in GPP are lacking. Currently investigated markers, including C-reactive protein (CRP) [113], primarily reflect disease activity rather than therapeutic response [114].

Notably, elevated serum IgE levels have also been observed in patients with GPP, despite the disease being classically associated with Th1/Th17 and innate immune pathways. Increased IgE levels correlate with systemic inflammatory markers such as CRP, suggesting that IgE may reflect overall inflammatory burden rather than a specific pathogenic pathway [115].

Emerging data suggest that plasma retinol levels may correlate with disease severity and response to acitretin, although these findings require further validation [116].

In addition to genetic determinants, circulating inflammatory mediators are being explored as potential biomarkers of disease activity in GPP. Serum amyloid A (SAA), an acute-phase protein involved in immune cell recruitment and cytokine induction, has been shown to be markedly elevated in patients with GPP compared with both plaque psoriasis and healthy controls. Notably, SAA levels correlate strongly with C-reactive protein and decrease in parallel with clinical improvement following biologic therapy, suggesting its potential utility as a dynamic biomarker of disease activity [82].

Additional circulating chemokines have also been investigated as potential biomarkers in GPP. Thymus and activation-regulated chemokine (TARC), a Th2-associated chemokine, has been shown to be significantly elevated in patients with GPP compared with plaque psoriasis and psoriatic arthritis. Importantly, serum TARC levels correlate with disease severity and decrease following treatment, suggesting their potential utility as a dynamic biomarker reflecting systemic inflammation and therapeutic response [83].

High-mobility group box 1 (HMGB-1), a proinflammatory damage-associated molecular pattern (DAMP), has also emerged as a potential biomarker in GPP. Both serum and skin levels of HMGB-1 are significantly elevated in patients with GPP compared with plaque psoriasis and other inflammatory dermatoses. Importantly, HMGB-1 levels correlate with disease severity and decrease following systemic treatment, supporting its potential role as a marker of disease activity and therapeutic response [84].

Data from a large retrospective cohort suggest potential for the development of clinically applicable biomarkers in GPP. Elevated direct bilirubin (DBIL > 8 µmol/L) has been proposed as a potential predictor of disease relapse, alongside clinical factors such as a history of plaque psoriasis. Importantly, treatment with biologics, particularly IL-36 inhibitors, was associated with significantly improved outcomes, including faster pustule resolution, reduced hospitalization, and a marked reduction in relapse risk [117].

Overall, most currently investigated serum biomarkers appear to reflect disease activity rather than reliably predict relapse risk, long-term disease course, or therapeutic response. In addition, their broader clinical applicability remains limited by insufficient validation, lack of standardized thresholds, variability across studies, and limited data regarding sensitivity, specificity, and longitudinal stability. Consequently, robust predictive biomarkers suitable for routine clinical practice are still lacking and remain a major unmet need in GPP research.

### 5.4. Neutrophil-Related Biomarkers and Differential Diagnosis

Neutrophil-derived biomarkers have also gained attention in the context of GPP. Neutrophil gelatinase-associated lipocalin (NGAL), a marker of neutrophil activation, has been shown to be significantly elevated in patients with GPP compared with AGEP, supporting its potential role in differential diagnosis. NGAL levels correlate with disease severity and decline following treatment, supporting their potential role as a dynamic marker of disease activity. In addition, NGAL may serve as an independent biomarker aiding in the differentiation of GPP from AGEP. Among currently investigated markers, NGAL appears particularly promising in differentiating GPP from AGEP; however, further validation in larger cohorts is required before routine clinical implementation and standardization [85].

Taken together, despite growing interest in biomarker development in GPP, no currently available biomarker demonstrates sufficient validation, predictive accuracy, and clinical applicability to support routine use in disease stratification, relapse prediction, or therapeutic decision-making. The identification of robust and clinically applicable predictive biomarkers therefore remains a major unmet need in GPP research.

### 5.5. Clinical Translation and Remaining Challenges of IL-36 Inhibition

These findings support the translational relevance of pathway-specific intervention in GPP. Notably, IL-36 inhibitors appear to achieve a faster onset of action compared with other biologics, with response rates such as GPPASI75 and GPPGA observed as early as 2 weeks, potentially outperforming TNF-α, IL-17, and IL-23 inhibitors during acute flares [87].

Nevertheless, the long-term safety and durability of response to IL-36 inhibitors in GPP remain important areas of ongoing investigation. Available data suggest that agents such as spesolimab are generally well tolerated and demonstrate a favorable safety profile, with no clear increase in serious adverse events compared with placebo, although infections and treatment-related immunogenicity have been reported. Maintenance therapy appears to reduce the risk of disease flares; however, relapses still occur in a subset of patients, indicating that sustained disease control remains challenging [87,92]. In addition, the currently available evidence remains limited by relatively small study populations, heterogeneous study designs, and the predominance of uncontrolled studies. Further longitudinal and real-world investigations are therefore needed to better define long-term safety, durability of response, optimal maintenance strategies, and flare-prevention protocols.

IL-36 inhibitors offer a dual advantage by enabling rapid control of acute disease activity while also supporting longer-term disease stability in patients prone to recurrence. These findings further support the rationale for mechanism-based therapeutic strategies aligned with the dominant inflammatory architecture of GPP [90,101,103,104].

## 6. Conclusions

Generalized pustular psoriasis is increasingly recognized as a distinct inflammatory disease with immunopathogenic features that differ substantially from those of plaque psoriasis. Accumulating evidence implicates dysregulated IL-36 signaling in GPP, although the disease remains clinically and molecularly heterogeneous. Comparative studies have demonstrated differences in inflammatory organization, transcriptomic signatures, genetic background, and therapeutic response, including distinct *IL36RN*-, *AP1S3*-, and *CARD14*-associated molecular pathways.

These differences have direct therapeutic implications. Conventional systemic therapies and biologics targeting TNF-α, IL-17, and IL-23 pathways continue to play an important role in selected clinical settings, particularly in maintenance therapy, concomitant plaque psoriasis, or where IL-36-targeted therapies are not readily available. However, therapies targeting the IL-36 pathway represent a highly promising mechanism-based approach and have demonstrated rapid and clinically meaningful efficacy in GPP, particularly during acute flares.

Despite these advances, major challenges remain. Although the IL-36 pathway represents a highly promising therapeutic target in GPP, predictive biomarkers capable of guiding patient selection, therapeutic response, relapse risk, and long-term disease monitoring are still lacking. In addition, currently available evidence is still derived largely from studies with limited sample sizes, heterogeneous designs, and insufficient long-term and real-world data. Further longitudinal investigations are needed to validate clinically applicable biomarkers, better characterize the clinical and molecular heterogeneity of GPP, clarify long-term safety and durability of response, and support the development of evidence-based maintenance treatment and flare-prevention strategies.

A deeper understanding of the interplay between innate and adaptive immune pathways, genetic susceptibility, and environmental triggers will be essential for advancing personalized therapeutic strategies and improving long-term disease control in patients with GPP. Taken together, current evidence supports the concept that GPP represents a distinct inflammatory disease with a unique immunopathogenic architecture. Further progress in biomarker development, longitudinal studies, and mechanism-based therapeutic approaches will be critical for improving disease stratification and optimizing long-term management in patients with GPP.

## 7. Materials and Methods

This work was designed as a narrative review based on a comprehensive analysis of the current literature concerning the role of the IL-36 signaling axis in GPP. Sections provide an in-depth review of studies retrieved from major biomedical and multidisciplinary databases, including PubMed, Google Scholar, Web of Science, Embase, and Scopus. The literature search was performed using combinations of the following keywords and Medical Subject Headings (MeSH): “generalized pustular psoriasis”, “GPP”, “IL-36”, “IL36RN”, “IL-36 signaling”, “innate immunity”, “neutrophils”, “plaque psoriasis”, “IL-23/Th17 axis”, “transcriptomic signatures”, “genetic mutations”, “spesolimab”, “imsidolimab”, “IL-36 inhibitors”, and “biomarkers in pustular psoriasis”. Figure 1 was created using BioRender.com.

The review focused primarily on publications from the last decade in order to reflect the most recent advances in the understanding of IL-36-driven inflammation and targeted therapies in GPP. However, earlier landmark studies published between 1909 and 2025 were also included to provide historical context and to capture pivotal discoveries regarding disease classification, immunopathogenesis, and genetic determinants.

Section 4 presents a critical appraisal of currently available therapeutic strategies for GPP, with particular emphasis on IL-36-targeted therapies and comparative limitations of conventional psoriasis biologics. This section includes an analysis of original clinical studies evaluating IL-36 receptor inhibitors, including spesolimab and imsidolimab. The selection of studies was based on a comprehensive structured literature search conducted primarily in PubMed and Scopus, focusing on original clinical trials, extension studies, and translational investigations involving patients with generalized pustular psoriasis. Review articles, conference abstracts without full data, and non-English publications lacking sufficient methodological detail were excluded to prioritize primary clinical evidence and mechanistic data.

## Figures and Tables

**Table 1:** Genetic determinants and associated clinical phenotypes in pustular psoriasis.

Gene	Gene Function	Age of Onset	Severity	Clinical Phenotype	Flare Characteristics	References
*IL36RN*	IL-36 receptor antagonist	Early (often childhood; variable)	Severe	GPP	Acute, recurrent, often systemic	[22,41,42]
*AP1S3*	Vesicular trafficking/autophagy regulation	Usually adults	Variable (moderate-severe)	PP	Recurrent	[42,43]
*CARD14*	NF-κB activation in keratinocytes	Variable (childhood to adulthood)	Variable	PSO ± PP	Episodic flares (trigger-dependent activation)	[44,45]

Abbreviations: GPP—generalized pustular psoriasis; PP—pustular psoriasis; PSO—plaque psoriasis; NF-κB—nuclear factor kappa-light-chain-enhancer of activated B cells.

**Table 2:** Comparative immunological, transcriptomic, genetic, and therapeutic features of plaque psoriasis and generalized pustular psoriasis.

Category	Plaque Psoriasis	Generalized Pustular Psoriasis	References
Dominant inflammatory axis	IL-23/Th17	IL-36	[53,54,58,60]
Age at onset	Bimodal onset (early adulthood and later adulthood)	Typically adulthood, peak 40–60 years	[1,4,5,6]
Flare pattern	Chronic, stable course	Relapsing disease with acute flares	[1,11,12]
Systemic symptoms	Typically absent	Common (fever, malaise, pain)	[1,21,24]
Laboratory markers	Elevated systemic inflammatory markers (CRP, IL-6, TNF-α)	Elevated CRP, leukocytosis, neutrophilia	[21,24,81]
Key immune drivers	IL-17A, IL-23, TNF, IFN-γ	IL-36, IL-1 family cytokines	[53,54,58,60]
Dominant cell types	T cells (Th17, Th1, Th22)	Neutrophils, keratinocytes	[53,54,58,60]
Transcriptomic signature	IL-17/TNF-driven genes	IL-36/MyD88 and IFN-I signatures	[64,66,67,68]
Genetic background	HLA-C*06:02, polygenic	*IL36RN*, *AP1S3*, *CARD14*	[22,43,44,75,76,77,78]
Candidate biomarkers	Limited clinically validated biomarkers	SAA, TARC, HMGB-1, NGAL (emerging biomarkers of activity)	[82,83,84,85,86]
Speed of therapeutic response	Gradual (weeks) with IL-17/IL-23 inhibitors	Rapid with IL-36 inhibitors (days–weeks); slower with conventional therapies	[55,56,57,87]
Therapeutic response	Strong response to IL-17/IL-23 inhibitors	Variable; IL-36 blockade emerging	[55,56,57,60,61]
Level of evidence for therapies	High (RCT-based evidence for IL-17/IL-23 inhibitors)	Limited overall; strongest for IL-36 inhibitors (RCTs), lower for others	[55,56,60,88,89,90]
Role of IL-36	Secondary amplifier	Central pathogenic pathway	[53,54,58,60]

Abbreviations: CRP—C-reactive protein, IL—Interleukin, TNF—Tumor necrosis factor, IL-17A—Interleukin-17A, IL-23—Interleukin-23, IFN-γ—Interferon gamma, IFN-I—Type I interferon, Th—T helper cell, MyD88—Myeloid differentiation primary response 88, HLA-C*06:02—Human leukocyte antigen C06:02, IL36RN—Interleukin-36 receptor antagonist gene, AP1S3—Adaptor-related protein complex 1 subunit sigma 3, CARD14—Caspase recruitment domain family member 14, SAA—Serum amyloid A, TARC—Thymus and activation-regulated chemokine, HMGB-1—High mobility group box 1, NGAL—Neutrophil gelatinase-associated lipocalin, RCT—Randomized controlled trial.

**Table 3:** Clinical response (GPPGA 0/1) in patients with GPP treated with spesolimab in a phase I clinical study.

Timepoint	Patients Achieving GPPGA 0/1, *n*
Week 1	5
Week 4	7

Abbreviations: GPP, generalized pustular psoriasis; GPPGA, Generalized Pustular Psoriasis Physician Global Assessment.

**Table 4:** Efficacy of spesolimab in the EFFISAYIL-1 trial after 1 week of treatment.

Endpoint	Spesolimab (*n* = 35)	Placebo (*n* = 18)
GPPGA 0	19/35 (54%)	1/18 (6%)
GPPGA 0/1	15/35 (43%)	2/18 (11%)

Abbreviations: GPPGA—Generalized Pustular Psoriasis Physician Global Assessment.

**Table 5:** Incidence of adverse events in the EFFISAYIL-1 trial.

Adverse Event, AE	Spesolimab, %	Placebo, %
Any AE	66%	56%
Infections	17%	6%
Asthenia/fatigue	9%	0%
Nausea/vomiting	6%	9%
Headache	9%	6%
Pruritus	6%	0%
Injection site hematoma/bruising	6%	0%

**Table 6:** Dosing regimens and efficacy outcomes in the EFFISAYIL-2 trial at Week 48.

Group	Dosing Regimen	Patients, *n*	Completed, *n*/*N*	Patients with ≥1 GPP Flare, %
High-dose spesolimab	600 mg SC loading dose, followed by 300 mg SC every 4 weeks	30	26/30	10%
Medium-dose spesolimab	600 mg SC loading dose, followed by 300 mg SC every 12 weeks	31	28/31	29%
Low-dose spesolimab	300 mg SC loading dose, followed by 150 mg SC every 4 weeks	31	27/31	23%
Placebo	Placebo SC every 4 weeks	31	30/31	52%

Abbreviations: SC—subcutaneous, GPP—generalized pustular psoriasis.

**Table 7:** Incidence of adverse events in the EFFISAYIL-2 trial.

Adverse Event, AE	Spesolimab, %	Placebo, %
Any AE	90%	87%
Severe adverse events	19%	23%
Serious adverse events	10%	3%
GPP flare	25%	53%
Psoriasis	14%	10%
Injection site erythema	14%	3%
Infections	33%	33%

**Table 8:** Comparison of IL-36 receptor inhibitors used in generalized pustular psoriasis.

Feature	Spesolimab	Imsidolimab
Study design	Phase I, II (EFFISAYIL-1, -2), ongoing extension (EFFISAYIL-ON); randomized, placebo-controlled	Phase II (GALLOP), Phase III (GEMINI-1, GEMINI-2); randomized, placebo-controlled
Key endpoints	GPPGA 0/1, time to flare, flare prevention	GPPGA 0/1, CGI response, maintenance of response
Time to response	Rapid (within 1 week)	Response observed within weeks (e.g., by week 4)
Safety	Generally favorable; mostly mild-to-moderate AEs	Favorable; low incidence of serious AEs
Immunogenicity	Anti-drug antibodies reported; unclear clinical impact	Low immunogenicity reported
Regulatory status	Approved (FDA, EMA) for GPP flares	Not yet approved
References	[24,90,92,103]	[96,108,109]

Abbreviations: AE—adverse event; CGI—Clinical Global Impression; EMA—European Medicines Agency; FDA—Food and Drug Administration; GPP—generalized pustular psoriasis; GPPGA—Generalized Pustular Psoriasis Physician Global Assessment.

## Data Availability

No new data were created or analyzed in this study.
